# Study on Non-Limited Earth Pressures of Soilbag-Reinforced Retaining Structures with Surcharge Loads

**DOI:** 10.3390/ma17030611

**Published:** 2024-01-26

**Authors:** Changrong Bian, Zhiqiang Lai, Ruonan Liu, Zhongmei Wang, Kewei Fan

**Affiliations:** 1 College of Civil and Transportation Engineering, Hohai University, Nanjing 210098, China; brian1997@hhu.edu.cn (C.B.); 211304010091@hhu.edu.cn (R.L.); 2 Yellow River Institute of Hydraulic Research, Yellow River Conservancy Commission, Zhengzhou 450003, China; wangzhongmei@hky.yrcc.gov.cn; 3 Key Laboratory of Lower Yellow River Channel and Estuary Regulation, Ministry of Water Resources, Zhengzhou 450003, China

**Keywords:** geosynthetics, soilbag, retaining wall, earth pressure, surcharge loads, non-limited state

## Abstract

The earth pressure acting on soilbag-reinforced retaining structures subjected to surcharge loads under non-limited states is crucial for designing these structures. In this study, mode tests on soilbag-reinforced retaining walls were conducted to the earth pressure of the wall subjected to surcharge loads. The findings from these tests reveal a non-linear distribution of lateral earth pressure on the wall when subjected to surcharge loads in non-limited states, with an observed escalation in pressure corresponding to increased surcharge loads. Insights from the tests facilitated the development of a predictive method for estimating lateral pressure on soilbag-reinforced retaining walls under similar conditions, and its performance was fully validated by the model tests. Furthermore, the impact of the geometric dimensions and material properties of the soilbags on the earth pressure distribution was examined using the proposed method.

## 1. Introduction

Geosynthetic materials, such as geotextiles, geomembranes, and geogrids, are often made from recycled or recyclable materials. Their use in civil engineering projects helps in reducing the dependency on natural resources and minimizes environmental footprints [1,2,3,4,5,6]. Soilbags are a form of application of geosynthetic materials in civil engineering. Traditionally, due to their limited durability, soilbags are primarily employed in constructing temporary embankments during floods and for building temporary structures in post-disaster scenarios [7]. However, following aging tests conducted by Matsuoka and Liu [8], it was asserted that soilbags exhibit enhanced durability when manufactured with anti-aging agents in woven bags. This advancement has led to the gradual adoption of these enhanced soilbags in permanent infrastructure projects, including reinforcing foundations [9], building retaining structures [10], and stabilizing slopes [11].

The soilbag-reinforced retaining structure is an important form in which soilbags are applied in engineering, and several application cases and the good performance of this new type of structure have been reported [12]. To establish well-supported design guidelines for scientifically engineering these structures, Fan et al. [10] explored the distribution of earth pressures behind soilbag-reinforced retaining walls through laboratory experiments and developed a method for analyzing the sliding stability of the wall based on the equilibrium of earth pressure and the interface resistance of soilbags. Wang et al. [13] investigated the effects of soilbag arrangements and tail length on the displacement and earth pressure of the wall. They proposed a method for estimating the wall’s safety using the upper-bound approach. Matsushima et al. [14] showcased many examples of soilbag-constructed retaining wall failure and found that one of the major drawbacks of this type of wall is the relatively low stability caused by slippage along the horizontal interface in between the adjacent soilbags, which results in a catastrophic failure. Addressing this, Liu et al. [15] conducted a series of lateral shear loading tests on a pile of three full-scale soilbags to examine the impact of inclined load on the shear strength of multi-layered soilbags. These studies collectively contribute valuable insights for designing soilbag-reinforced retaining structures.

In a large number of retaining structure projects, additional surcharge loads, such as those from vehicles and buildings, are often present on the surface of the backfill behind the structures. These loads negatively impact the stability of the retaining structure [16,17,18,19,20]. It is therefore essential to determine the earth pressure exerted on soilbag-reinforced retaining walls when subjected to such surcharge loads. However, existing research has primarily focused on soilbag-reinforced retaining walls under limit states [10,13]. In practical engineering situations, this state of limit equilibrium is often not reached, resulting in a non-limited state for the wall [21,22]. This divergence is attributed to factors such as the restricted movement of soilbags and the complex behavior of soil. As a result, there is a need for a more sophisticated approach to accurately estimate the active earth pressure on soilbag-reinforced retaining walls under surcharge loads in a non-limited state, to better mirror the actual conditions faced by these structures.

In this study, physical model tests were performed to investigate the earth pressure exerted on the soilbag-reinforced retaining wall subjected to surcharge loads under the non-limited state. To predict the non-limited lateral earth pressure, an analytical method grounded in the force equilibrium of differential elements was formulated. The validity and effectiveness of this analytical approach were subsequently corroborated by the results derived from these model tests. The effect of the geometric size and material properties of the soilbags on the distribution of the earth pressure is also analyzed using this method. This study contributes to the application of geosynthetic materials in civil and geological engineering. The main contribution of this study to the *Materials* journal is the development and validation of a new method for accurately predicting lateral earth pressure on soilbag-reinforced retaining walls under surcharge loads. This work enhances understanding of earth pressure distribution and provides a practical tool for the design and safety of these structures, addressing a significant gap in the field of geotechnical engineering.

## 2. Experimental Investigations

### 2.1. Model Test

**Figure 1:**
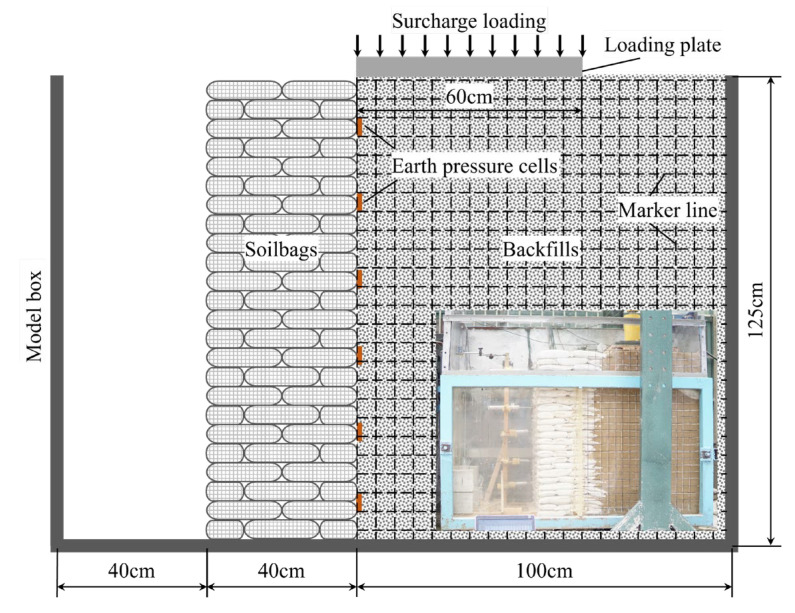
Schematic diagram of soilbag-reinforced retaining wall.

Model tests on a soilbag-reinforced retaining wall were conducted in a rectangular box measuring 1.8 m in length, 0.8 m in width, and 1.25 m in height, as depicted in Figure 1. For observation, a 2 cm thick glass sheet, resistant to deformation, was installed on the box’s front face. The tests involved a soilbag-reinforced retaining wall of 80 cm length, 40 cm width, and 120 cm height, constructed using two staggered sizes of soilbags: one measuring 20 cm × 20 cm × 5 cm and the other 20 cm × 10 cm × 5 cm. These soilbags, fabricated from woven bags weighing 150 g/m^2^, are detailed in Table 1 regarding their main performance parameters. The bags were filled with river sand from Nanjing, China, which was also used as the backfill soil for the wall, with a density of 1.76 g/cm^3^. The maximum and minimum dry densities of the sand were 1.66 g/cm^3^ and 1.89 g/cm^3^, respectively, with an unevenness coefficient of *C*_u_ = 2.0, a curvature coefficient of *C*_c_ = 1.28, and an internal friction angle of 35.4°. Behind the wall, a 1 m wide backfill section was established, with a 0.7 m wide loading plate positioned near the wall’s top. This plate, narrower than the box width (0.8 m), facilitated ease of insertion and minimized friction between the plate and the box. The surcharge load applied on the wall was incrementally increased until the wall’s failure was observed.

For the evaluation of the model testing wall’s response to surcharge loads, six small earth pressure cells, each measuring 2 cm in diameter and 0.5 cm in height, were strategically embedded in the sand during its placement, as depicted in Figure 1. These cells, capable of measuring pressures in the range of 0–50 kPa with a precision of up to 0.1 kPa, were essential for accurate data collection. To facilitate the observation of backfill movement, a series of black marker lines were applied to the external surface of the glass, complemented by an equal number of white cotton lines aligned internally in identical positions. These internal lines were designed to shift corresponding to the movement of the backfill, whereas the external marker lines were intended to remain fixed. This arrangement allowed for the determination of backfill movement through the comparison of the relative positions of these marker lines, both inside and outside the glass. To systematically monitor these movements, a camera was strategically positioned in front of the model setup, enabling the continuous tracking of line movement at predetermined intervals.

### 2.2. Test Result

**Figure 2:**
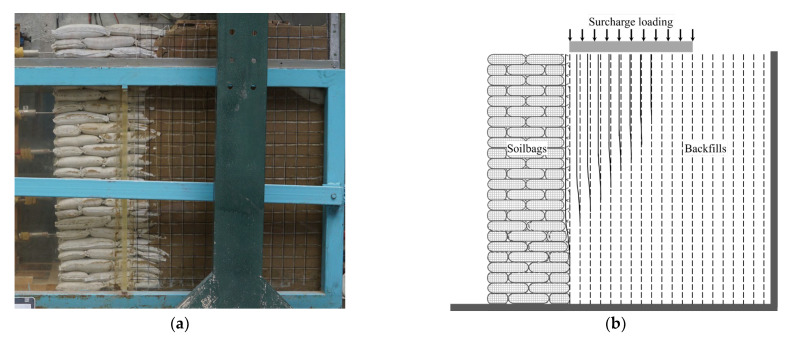
Deformation of the backfill in the wall subjected to surcharge stress of 4 kPa (**a**) photo; (**b**) schematic diagram.

**Figure 3:**
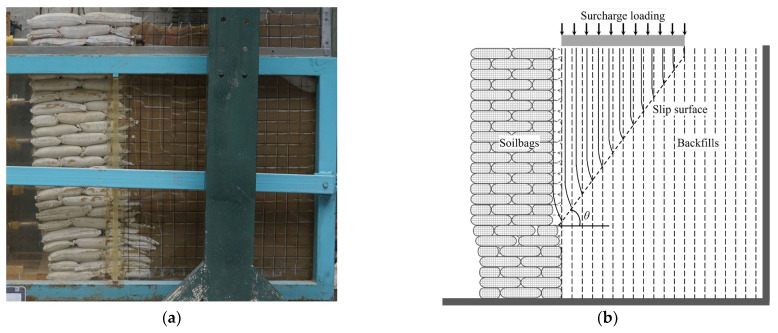
Deformation of the backfill in the wall subjected to surcharge stress of 8.7 kPa (**a**) photo; (**b**) schematic diagram.

In the conducted model test, the retaining wall experienced failure when the surcharge stress escalated to 8.7 kPa. Figure 2 and Figure 3 depict the deformation of white cotton lines at surcharge stresses of 4 kPa and 8.7 kPa, respectively. It was observed that at a surcharge stress of 4 kPa, the backfill exhibited movement toward the soilbag-reinforced retaining wall, as indicated by the displacement of the white cotton lines relative to the black marking lines. This movement was attributed to the compression deformation of the soilbag under the surcharge stress. Upon reaching a surcharge stress of 8.7 kPa, a distinctive stepped sliding surface emerged within the structure of the soilbag-reinforced retaining wall, signaling its failure, as evidenced in Figure 3. Fan et al. [10] claimed that the ladder-like sliding surface formation is attributed to the interlayer insertion of soilbags. When the wall failed, a sliding surface emerged in the backfill behind the wall, which aligned nearly in a straight line. This sliding surface formed an angle of 60° with the horizontal plane, closely resembling the theoretical angle of 45 degrees plus half the internal friction angle (*φ*/2), as illustrated in Figure 3b. The upper portion of this stepped sliding surface exhibited horizontal movement. The critical height of the sliding wall (*H*_crit_) in this scenario was measured at 0.95 m.

**Figure 4:**
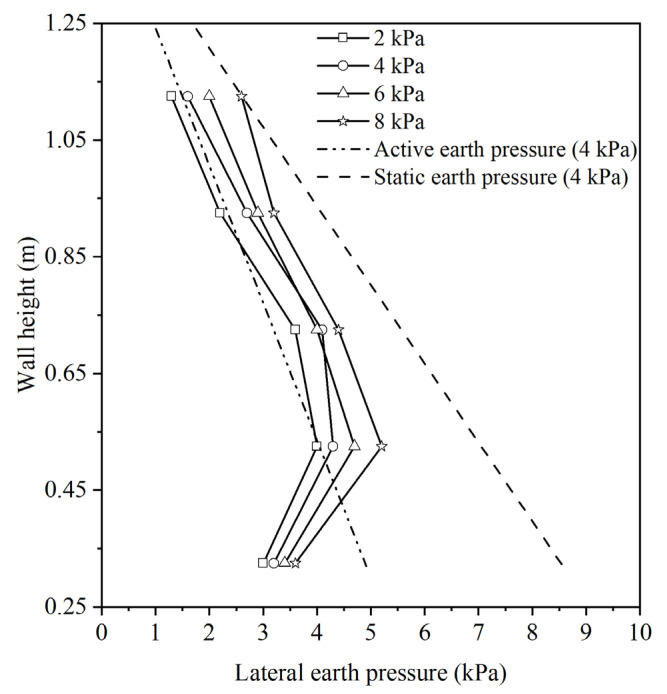
Distribution of lateral earth pressures on soilbag-reinforced retaining walls subjected to surcharge loads under non-limited states.

This present research focuses on assessing the earth pressure on walls subjected to surcharge loads under non-limited conditions. Therefore, the lateral earth pressure acting on the wall at different stages of surcharge stress, specifically at 2 kPa, 4 kPa, 6 kPa, and 8 kPa, were measured. The results are shown in Figure 4. Notably, during these stages, the retaining wall remains undamaged. The distribution of lateral earth pressure on the wall is characterized as non-linear. As the surcharge stress increases, there is a corresponding increase in the lateral earth pressure exerted on the wall. The static and active earth pressures of the wall at the surcharge stress of 4 kPa were calculated using Coulomb’s earth pressure theory, as illustrated in Figure 4. The earth pressures acting on the wall were found to be within the range of the static and active earth pressures, confirming the non-limited state of the earth pressure.

## 3. Analytical Approaches

The assessment of earth pressure is a crucial component in studies focusing on retaining structures, as it significantly influences both the stability and the cost of retaining walls. Within this realm, the Rankine and Coulomb earth pressure theories are recognized as foundational approaches for estimating earth pressure. However, a limitation shared by these theories is their assumption of soil remaining static, disregarding any wall displacement. Through comprehensive model experiments, the active earth pressure displays a nonlinear pattern when retaining walls experience either translation or rotation, thereby rendering traditional earth pressure theories inadequate [23]. Consequently, numerous academics have developed alternative approaches for calculating earth pressure under varying displacement scenarios of retaining walls. Widely adopted methods include the differential element method [24,25,26] and the finite element numerical method [27,28,29]. The differential element method, specifically, is formulated based on conditions involving a retaining wall with a vertical back, sandy soil fill, and a horizontal surface behind the wall. This method segments the sliding wedge behind the wall into horizontal soil elements, presuming a uniform vertical stress distribution across these elements. Subsequently, the earth pressure calculation solution is derived by considering both the static and moment equilibrium of the soil elements, revealing a nonlinear soil pressure distribution. This method has been effectively applied in calculating active earth pressure for various types of retaining walls, yielding favorable results [30,31,32].

Originally, the differential element method was utilized predominantly for calculating the active earth pressure of retaining walls in a limited state. To extend its application to non-limited states, several scholars have refined this approach. Notably, Tang and Chen [33] contributed by developing theoretical formulations linking wall displacement to the friction angle in non-limited states. Subsequent methodologies introduced by other researchers have largely followed the foundational principles set by Tang and Chen, with variations in computing the effective friction angle under non-limited state scenarios [34].

In the conducted model test, it was observed that the active earth pressure exerted on the soilbag-reinforced retaining wall under non-limited conditions displayed a nonlinear behavior. Furthermore, the conditions under which the wall operated were consistent with the prerequisites for applying the differential element method. As a result, this method was effectively employed to compute the non-linear earth pressures that were recorded in the test.

### 3.1. Basic Equation for Non-Limited Active Earth Pressure

**Figure 5:**
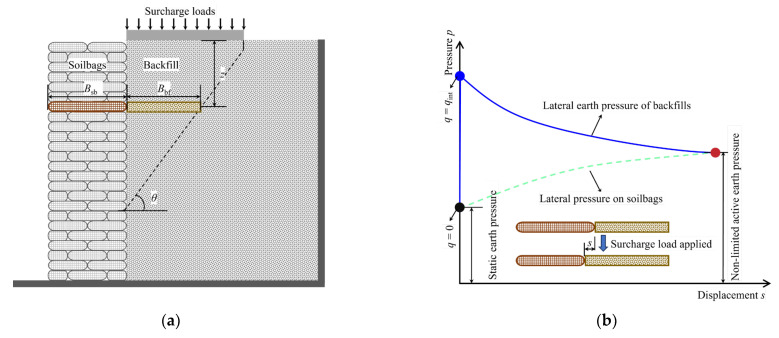
Analytical model: (**a**) differential flat element of backfill in sliding wedge; (**b**) variations of lateral earth pressure caused by backfill and lateral pressure on soilbags.

In the context of analyzing a sliding wedge as an isolated entity, as depicted in Figure 5a, a differential flat element with a thickness of *dz* is selected from the wedge, positioned at a depth of *z* beneath the backfill surface. In conditions where surcharge stress is absent (*q* = 0), the pressure exerted by the backfill due to filling is characterized as static earth pressure. Conversely, when surcharge stress, which does not compromise the integrity of the retaining wall (*q* = *q*_int_), is applied to the backfill, the soil’s immediate response is hindered, leading to a swift increase in lateral earth pressure, as demonstrated in Figure 5b. This escalation in lateral earth pressure consequently prompts compression in the neighboring soilbag, resulting in augmented compression deformation of the soilbag. Concurrently, there is an observable increase in the swelling deformation along with a decrease in the backfill’s lateral earth pressure. Equilibrium is attained once the lateral earth pressure equilibrates with the lateral compressive stress applied to the soilbag. At this juncture, the lateral swelling deformation of the backfill matches the lateral compressive deformation of the soilbag, as shown in Figure 5b. The relationship established upon achieving equilibrium following the application of surcharge stress *q*_int_ is as follows:

(1) $$p_{x-bf}=\sigma_{x-sb}\;\varepsilon_{x-bf}B_{bf}=\varepsilon_{x-sb}B_{sb}$$

where *p*_x−bf_ and *ε*_x−bf_ are the lateral earth pressure and strain of the backfill, *σ*_x−sb_ and *ε*_x−sb_ are the lateral compressed stress and strain of soilbag, *B*_bf_ is the width of the differential flat element, and *B*_sb_ is the width of the soilbagreinforced retaining wall.

Building upon the findings of Wang [35], the lateral earth pressure exerted by the backfill can be expressed as:

(2) $$p_{x-bf}=K_{q}q+K_{\gamma }\gamma z$$

where *K*_q_ and *K*_γ_ are the surcharge pressure coefficient and the backfill pressure coefficient, respectively. These coefficients are inherently linked to the strain experienced by the differential flat element.

The lateral compressed stress of soilbags can be expressed as:

(3) $$\sigma_{x-sb}=E_{x-sb}\varepsilon_{x-sb}+K_{0}\gamma z$$

where *E*_x−sb_ is the elastic modulus of the soilbag, and *K*_0_ is the static earth pressure coefficient of the backfill.

By solving simultaneous Equations (1)–(3), the non-limited active earth pressure exerted on soilbag-reinforced retaining walls subjected to surcharge loads can be determined.

### 3.2. Determination of K_q_ and K_γ_

In limited-state scenarios, retaining walls exhibit complete sliding wedges, where the backfill’s internal friction angle (*φ*_cirt_) and the friction angle at the backfill-wall interface (*δ*_crit_) peak. In contrast, non-limited states yield only partial wedge development, resulting in lower, or intermediate, friction angles (*φ*_int_ for backfill internal friction and *δ*_int_ for backfill-wall friction). These intermediate values, lower than *φ*_cirt_ and *δ*_crit_, indicate a less intense stress condition. Fan et al. [36] outline a procedure to calculate *φ*_int_ and *δ*_int_, suggesting these angles lie between their initial (*φ*_0_ and *δ*_0_, representing the starting internal and interface friction angles) and critical states. This approach helps in precisely gauging the frictional behavior of retaining walls under varying conditions.

The calculation equations provided by Fan et al. [36] for *φ*_int_ and *δ*_int_ are as follows:

(4) $${\tan\varphi }_{int}={\tan\varphi }_{0}+\left({\tan\varphi }_{crit}-{\tan\varphi }_{0}\right)\frac{s}{s_{crit}}\;(0\leq s<s_{crit})\;{\tan\varphi }_{int}={\tan\varphi }_{crit}\;(s\geq s_{crit})$$

(5) $${\tan\delta }_{int}={\tan\delta }_{0}+\left({\tan\delta }_{crit}-{\tan\delta }_{0}\right)\frac{s}{s_{crit}}\;(0\leq s<s_{crit})\;{\tan\delta }_{int}={\tan\delta }_{crit}\;(s\geq s_{crit})$$

where *s*_crit_ is the critical lateral displacement necessary to reach the fully active state of the backfill. For walls with layered backfill, *δ*_0_ is proposed to be half of *φ*_cirt_ (i.e., *δ*_0_ = *φ*_cirt_/2), and *φ*_0_ can be derived from Equation (6) [37]:

(6) $${\left(\frac{1}{{\cos\varphi }_{0}}+\sqrt{{\tan^{2}\varphi }_{0}+{\tan\varphi }_{0}{\tan\varphi }_{0}}\right)}^{2}=\frac{1}{1-{\sin\varphi }_{crit}}$$

Sherif et al. [38] provided an empirical equation for *s*_crit_ in retaining walls with varying densities of sand through model tests:

(7) $$s_{crit}=H_{crit}(7.0-0.13{\sin\varphi }_{crit})(10^{-4})$$

By solving Equations (4)–(7), the values of *φ*_int_ and *δ*_int_ can be determined.

From the equilibrium analysis of horizontal and vertical forces, as well as the moment equilibrium about the midpoint on the sliding surface of a differential flat element, a set of equations can be derived:

1. Equation (8) represents the balance of horizontal forces:

(8) $$p_{x-bf}{\cos\delta }_{int}dz-R_{bf}cos(90-\theta +\varphi_{int})dz/sin\theta =0$$

2. Equation (9) addresses the vertical force equilibrium, given by:

(9) $$\begin{array}{c} q_{\mathrm{bf}}\left(H_{\mathrm{crit}}-z\right)\cot\theta -\left(q_{\mathrm{bf}}+dq_{\mathrm{bf}}\right)\left(H_{\mathrm{crit}}-z-dz\right)\cot\theta +dW_{\mathrm{bf}}\\ \;\;\;\;\;-p_{x-\mathrm{bf}}\sin\delta_{\mathrm{int}}dz-R_{\mathrm{bf}}\cos\left(90-\theta +\varphi_{\mathrm{int}}\right)dz/\sin\theta =0 \end{array}$$

3. Equation (10) pertains to the moment equilibrium:

(10) $$\begin{array}{cc} p_{x-\mathrm{bf}}\left(H_{\mathrm{crit}}-z-\right. & dz/2)\cot\theta \sin\delta_{\mathrm{int}}dz\\ \; & -q_{\mathrm{bf}}\left(H_{\mathrm{crit}}-z\right)\cot\theta \left(H_{\mathrm{crit}}-z-dz\right)\cot\theta /2\\ \; & +\left(q_{\mathrm{bf}}+dq_{\mathrm{bf}}\right)\left(H_{\mathrm{crit}}-z-dz\right)\left(H_{\mathrm{crit}}-z\right)\cot\theta /2\\ \; & -\gamma \left(H_{\mathrm{crit}}-z-dz\right)\cot\theta \left(H_{\mathrm{crit}}-z\right)\cot\theta dz/2=0 \end{array}$$

In these equations, *θ* is the sliding angle (*θ* = 45° + φ/2), *q*_bf_ is the force on the top of the element, *R*_bf_ is the normal reaction of the backfill soil at rest, and *dW* is the weight of the element (*dW*_bf_ = *γ* (*H*_crit_ − *z*) cot *θ* d*z*).

Equations (8) and (10) can be simplified as:

(11) $$R_{bf}=p_{x-bf}\frac{{\cos\delta }_{int}sin\theta }{sin(\theta -\varphi_{int})}$$

(12) $${dq}_{bf}=\gamma dz-2p_{x-bf}\tan\theta \;{\sin\delta }_{int}dz/\left(H_{\mathrm{crit}}-z\right)=0$$

Ignoring the second-order differential terms yields, Equation (9) can be expressed as:

(13) $$\begin{array}{c} dq_{\mathrm{bf}}=\gamma dz+q_{\mathrm{bf}}dz/\left(H_{\mathrm{crit}}-z\right)\\ \;\;\;\;\;-p_{x-\mathrm{bf}}\tan\theta \sin\delta_{\mathrm{int}\;}\left[1+\cot\delta_{\mathrm{int}}\cot\left(\theta -\varphi_{\mathrm{int}}\right)\right]dz/\left(H_{\mathrm{crit}}-z\right) \end{array}$$

Substituting Equation (12) into Equation (13) yields

(14) $$q_{bf}=\tan\theta \;{\sin\delta }_{int}\left({\cot\delta }_{int}\cot\left(\theta -\varphi_{int}\right)-1\right)p_{x-bf}$$

Substituting Equation (14) into Equation (12) yields:

(15) $$\frac{{dq}_{bf}}{dz}=-\frac{2Nq_{bf}}{\left(H_{\mathrm{crit}}-z\right)}+\gamma$$

where *N* = 1/(cot *δ*_int_ cot (*θ − φ*_int_) − 1).

By differentiation, the general solution of Equation (15) is

(16) $$q_{bf}=C{\left(H_{\mathrm{crit}}-z\right)}^{2N}+\frac{\gamma }{2N-1}\left(H_{\mathrm{crit}}-z\right)$$

where *C* is a constant, which can be determined by the boundary condition (*z* = 0, *q*_bf_ = *q*). *C* is determined as

(17) $$C=\left(q-\frac{\gamma H_{\mathrm{crit}}}{2N-1}\right){H_{\mathrm{crit}}}^{-2N}$$

Thus, Equation (16) can also be expressed as:

(18) $$q_{bf}=\left(q-\frac{\gamma H_{\mathrm{crit}}}{2N-1}\right){\left(\frac{H_{\mathrm{crit}}-z}{H_{\mathrm{crit}}}\right)}^{-2N}+\frac{\gamma }{2N-1}\left(H_{\mathrm{crit}}-z\right)$$

Substituting Equation (18) into Equation (14), *p*_x−bf_ can be obtained:

(19) $$\begin{array}{c} p_{x-\mathrm{bf}}=\frac{N}{\tan\;\theta \;\sin\;\delta_{\mathrm{int}\;}}{\left(\frac{H_{\mathrm{crit}\;}-Z}{H_{\mathrm{crit}\;}}\right)}^{2N}q\\ \;\;\;\;\;\;-\frac{N}{\left(2N-1\right)\;\tan\;\theta \;\sin\;\delta_{\mathrm{int}\;}}\frac{H_{\mathrm{crit}\;}-Z}{Z}\left[1-{\left(\frac{H_{\mathrm{crit}\;}-Z}{H_{\mathrm{crit}\;}}\right)}^{2N-1}\right]\gamma Z \end{array}$$

Solving for Equations (2) and (19) yields:

(20) $$K_{q}=\frac{N}{\tan\theta \sin\delta_{\mathrm{int}}\left(\cot\delta_{\mathrm{int}}\cot\left(\theta -\varphi_{\mathrm{int}}\right)-1\right)}{\left(\frac{H_{\mathrm{crit}\;}-z}{H_{\mathrm{crit}\;}}\right)}^{2\left(\cot\delta_{\mathrm{int}\;}\cot\left(\theta -\varphi_{\mathrm{int}\;}\right)-1\right)}$$

(21) $$\begin{array}{c} K_{\gamma }=\frac{H_{\mathrm{crit}\;}-z}{z\;\left(3\;-\;\cot\delta_{\mathrm{int}\;}\cot\left(\theta -\varphi_{\mathrm{int}\;}\right)\right)\tan\;\theta \;\sin\;\delta_{\mathrm{int}\;}}[1\\ \;\;\;\;\;\;-{\left(\frac{H_{\mathrm{crit}\;}-z}{H_{\mathrm{crit}\;}}\right)}^{2\cot\delta_{\mathrm{int}\;}\cot\left(\theta -\varphi_{\mathrm{int}\;}\right)-3}] \end{array}$$

### 3.3. Determination of E_x−sb_

The lateral compressive stress–strain characteristics of soilbags were investigated using a direct shear test apparatus, details of which are elaborated in the work of Fan et al. [5]. In this setup, two layers of soilbags were positioned on the apparatus’s base. The lower layer was anchored using two plates fixed to the base, ensuring stability during testing. Above the soilbags, a loading plate was placed, accompanied by a right-side plate. This arrangement was designed to apply normal stresses to the soilbags. A critical component of the setup was a displacement sensor attached to the right-side plate. This sensor’s role was to precisely track any lateral displacement that occurred during the testing process. For the application of lateral force, a controlled speed of 1 mm/min was maintained, and a load cell was incorporated to measure the lateral force exerted.

**Figure 6:**
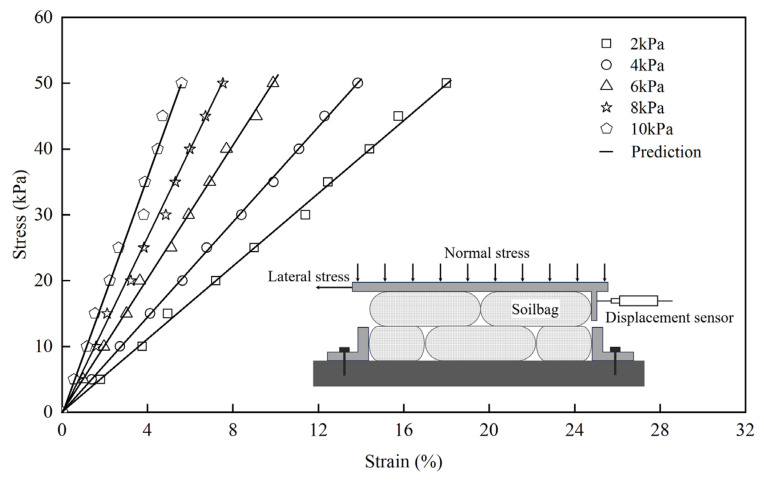
Lateral stress–strain relationship of soilbags at different normal stresses.

**Figure 7:**
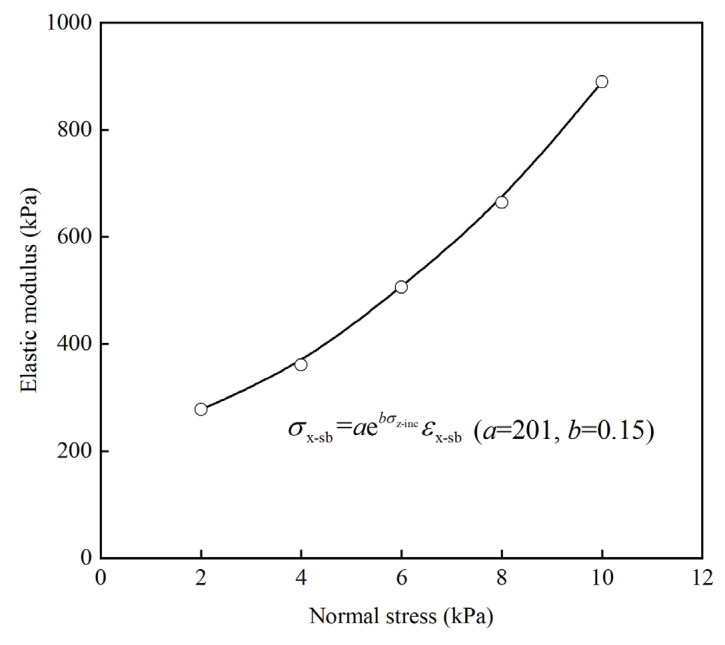
Relationship between elastic modulus and normal stresses of soilbags.

The results of these tests are depicted in Figure 6, which illustrates the lateral compressive stress–strain relationship of the soilbags under varying normal stresses. The data indicate a roughly linear relationship between lateral stress and strain across different levels of normal stress. This linear trend suggests a consistent response of the soilbags to lateral compression. Figure 7 presents the correlation between the lateral compressive modulus (*E*_x−sb_) and the normal stress (*σ*_z−sb_) of the soilbag. This relationship reveals that the *E*_x−sb_ value progressively increases as *σ*_z−sb_ increases, demonstrating a distinct exponential connection between these two parameters. This observed relationship can be quantitatively described by an exponential function, where the lateral stress–strain relationship of the soilbag is modeled as:

(22) $$\sigma_{x-sb}=201e^{0.15\sigma_{z-sb}}\varepsilon_{x-sb}$$

Further simplification leads to the expression for the lateral compressive modulus (*E*_x−sb_) as:

(23) $$E_{x-sb}=ae^{b\sigma_{z-sb}}$$

where *a* = 201 and *b* = 0.15.

### 3.4. Validation

In the described model test, key parameters include the unit weight of the backfill (*γ*) at 17.6 kN/m^3^, the internal friction angle (*φ*_crit_) at 35.4°, the frictional angle between the back of the soilbag and the backfill (*δ*_crit_) at 28.1°, the width of the soilbag-reinforced retaining wall (*B*_sb_) at 0.4 m, and the critical height of the wall (*H*_crit_) at 0.95 m. Utilizing the previously mentioned equations, the lateral displacement and lateral earth pressures of the backfill along the wall height were calculated under varying surcharge loads of 2 kPa, 4 kPa, 6 kPa, and 8 kPa.

**Figure 8:**
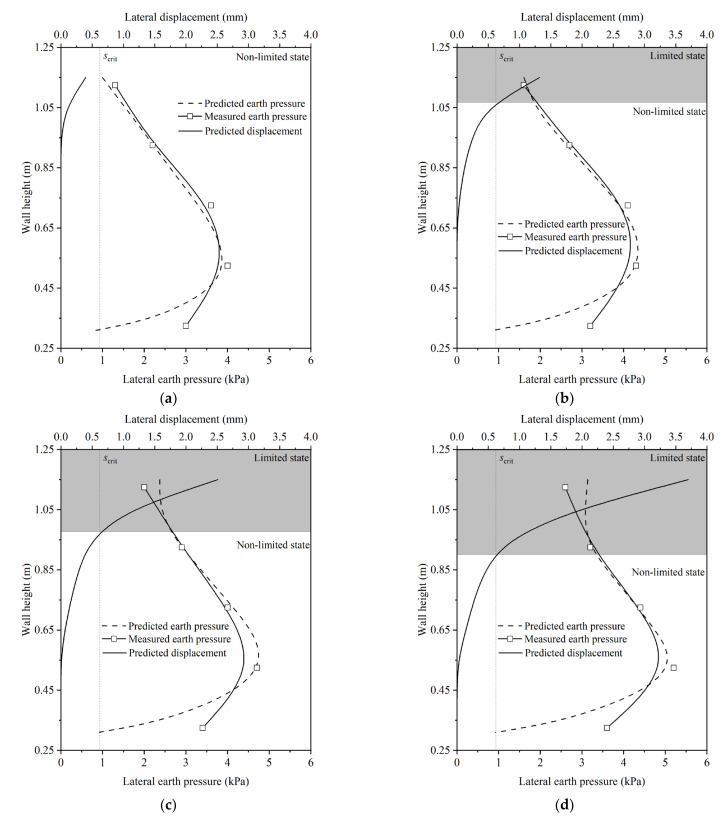
Distribution of non-limited lateral earth pressures under different surcharge loads of (**a**) 2 kPa, (**b**) 4 kPa, (**c**) 6 kPa, and (**d**) 8 kPa.

The findings revealed that under a 2 kPa surcharge stress, the calculated lateral displacement along the wall height remained below the critical lateral displacement (*s*_crit_). This suggests that, in this scenario, the wall did not reach its limit state, as depicted in Figure 8a. However, as the surcharge load increased to 4 kPa, a notable change was observed. The lateral displacement of the soilbag at the upper part of the wall exceeded *s*_crit_, indicating that this portion of the wall had reached the limit state, as shown in Figure 8b. Furthermore, the extent of the wall reaching the limit state increased progressively with the increment of the surcharge loads, as shown in Figure 8c,d. Another key observation from Figure 8a–d is the close agreement between the calculated earth pressure and the experimental data. This alignment validates the accuracy of the calculations and the effectiveness of the equations used in predicting the behavior of the soilbag and the retaining wall under different loading conditions.

### 3.5. Influencing Factors Analysis

**Figure 9:**
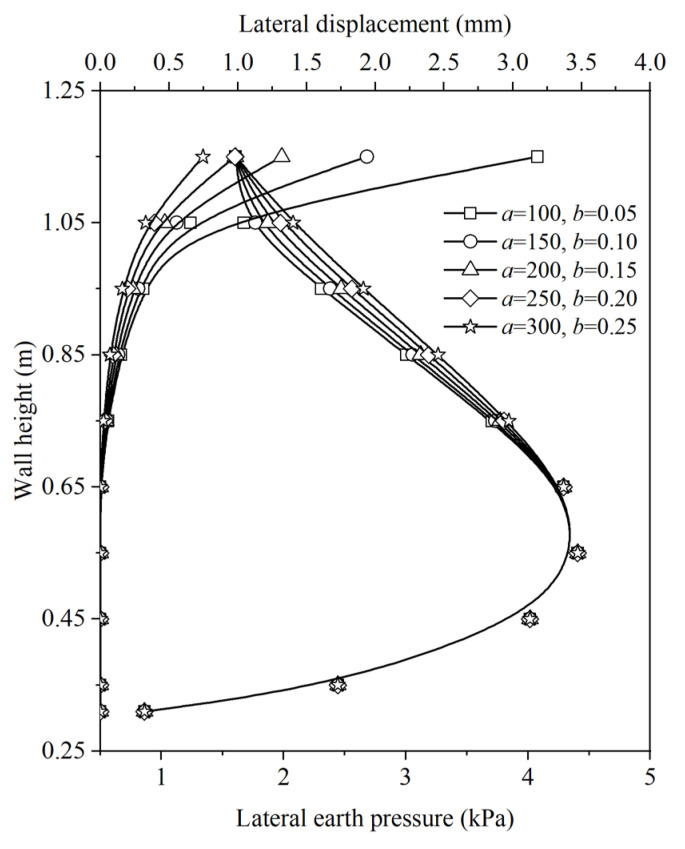
Effect of *E*_x−sb_ on distributions of lateral displacement and earth pressure along the wall height.

**Figure 10:**
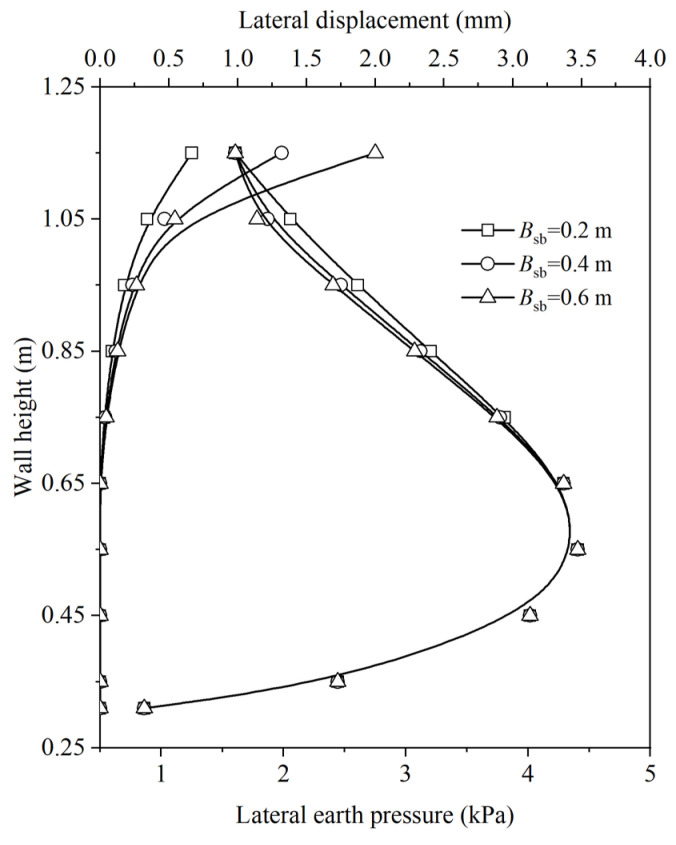
Effect of *B*_sb_ on distributions of lateral displacement and earth pressure along the wall height.

The effect of soilbag-related parameters, namely the compression modulus of the soilbag (*E*_x−sb_) and the width of the soilbag-reinforced retaining wall (*B*_sb_), on the distributions of lateral displacement and earth pressure along the wall height at a surcharge stress of 4 kPa, was investigated using the proposed method. Equation (23) reveals a positive correlation between the compression modulus of the soilbag and parameters *a* and *b*. By varying the values of *a* and *b*, the impact of *E*_x−sb_ on earth pressure and deformation was explored, as illustrated in Figure 9. An increase in *E*_x−sb_ was observed to reduce the lateral displacement caused by the soilbag. This reduction in lateral displacement of the backfill led to an increase in earth pressure. The effect of varying *B*_sb_ on earth pressure and deformation is presented in Figure 10. Theoretically, augmenting the wall’s thickness is posited to enhance its stability. However, under a specific surcharge load, the earth pressure is observed to decrease with an increase in wall thickness, while lateral displacement exhibits an increase with wall thickening.

## 4. Conclusions

Model tests on the soilbag-reinforced retaining wall subjected to surcharge loads under the non-limited state were carried out to analyze the non-limited earth pressures. The findings yielded several key insights:

1. The lateral earth pressure distribution on the soilbag-reinforced retaining wall subjected to surcharge loads under the non-limited state is non-linear. As the surcharge loads increased, there was a corresponding increase in the earth pressure.
2. It was determined through experimentation that the lateral stress–strain behavior of the soilbags adheres to a linear model. Furthermore, an exponential relationship between the lateral compression modulus of the soilbags and the normal stress was established.
3. The analytical solution, developed based on the force equilibrium of differential elements, proved to be highly effective in predicting the non-limited lateral earth pressure on the soilbag-reinforced retaining wall under surcharge loads. The effect of the compression modulus of the soilbag and the width of the soilbag-reinforced retaining wall on the non-limited earth pressure was examined using the analytical solution. It is shown that the earth pressure increases with the increasing compression modulus of the soilbag and the decreasing width of the wall.

Although the calculated earth pressure aligns well with the experimental data, aspects such as arching effects and their influence on earth pressure, which have been acknowledged to affect earth pressure in previous studies, were not investigated in this research. Additionally, the scope of our experimental setup and the scale of the model tests may limit the direct applicability of our findings to real-world scenarios without further validation. It is advised that readers consider these limitations while interpreting the findings.

## Figures and Tables

**Table 1:** Main performance parameters of woven bags.

Raw Materials	Mass per Unit Area (g/m^2^)	Tensile Strength (kN/m)		Elongation (%)		Friction Coefficient
		Warp	Weft	Warp	Weft	
Polypropylene	150	37.1	28.0	13.7	15.9	0.54

## Data Availability

The data presented in this study may be available on reasonable request from the first or corresponding author.
